# Therapeutically relevant rimonabant exposure drives epigenetic remodeling in neuronal cells and rat brain tissue

**DOI:** 10.1007/s00204-026-04369-0

**Published:** 2026-04-17

**Authors:** Sandra I. Marques, Matilde Barreiras de Moura, Federica Panza, Catarina Pereira-Teixeira, Vladimir Stevanović, Aleksandra Kovačević, Miroslav M. Savić, Helena Carmo, Susana I. Sá, Félix Carvalho, João Pedro Silva

**Affiliations:** 1 UCIBIO – Applied Molecular Biosciences Unit, Laboratory of Toxicology, Faculty of Pharmacy, University of Porto, 4050-313 Porto, Portugal; 2 i4HB – Institute for Health and Bioeconomy, Faculty of Pharmacy, University of Porto, 4050-313 Porto, Portugal; 3 Department of Pharmacology, Faculty of Pharmacy, University of Belgrade, Belgrade, Serbia; 4 RISE-HEALTH, Faculty of Medicine, University of Porto, Porto, Portugal

**Keywords:** SR141716A, Histone acetylation, Histone methylation, Psychiatric and psychological adverse events, DNA methylation

## Abstract

Rimonabant (SR141716A), an inverse agonist of the cannabinoid receptor type 1 (CB_1_), once approved for treating obesity and metabolic disorders, was withdrawn shortly after due to psychiatric and psychological adverse events (PPAEs), including depression and suicidality. Although its primary pharmacological mechanism of action is well-characterized, the molecular basis underlying these neuropsychiatric effects remains unclear. Here, we investigated the epigenetic impact of rimonabant exposure, both in vitro and ex vivo, at therapeutically relevant concentrations and doses, with a focus on histone modifications and DNA methylation. In SH-SY5Y human neuroblastoma cells, after 24 and 96 h treatment with 0.01 and 1 µM rimonabant significantly increased global histone H3 and H4 acetylation by 2.7- and 1.4-fold, respectively, without altering global DNA methylation levels. The effects on histone acetylation were partially reversed by a CB_1_ receptor agonist, indicating a role for CB_1_ in the observed epigenetic modulation. Rimonabant also decreased histone deacetylases (HDAC) activity and reduced the levels of H3K4me3 and H3K27me3, marks that have been previously identified in psychiatric perturbations. Moreover, 4-week oral administration of 3 or 15 mg/kg rimonabant to rats produced region- and dose-specific alterations in H3K4me3, H3K27me3, H3K9ac, and 5-methylcytosine levels across the prefrontal cortex, hippocampus, and nucleus accumbens, in line with epigenetic profiles characteristic of depression, anxiety, and schizophrenia. Collectively, these findings demonstrate that rimonabant disrupts key epigenetic regulatory mechanisms in the brain and support the hypothesis that epigenetic dysregulation contributes to its psychiatric liabilities. This work strengthens the value of incorporating epigenetic endpoints into neuropharmacological safety assessments.

**Supplementary Information:**

The online version contains supplementary material available at 10.1007/s00204-026-04369-0.

## Introduction

Numerous pharmaceuticals, spanning diverse classes (e.g., selective serotonin reuptake inhibitors, fluoroquinolones, monoclonal antibodies, hormonal therapies) have been associated with the onset of psychiatric/psychological adverse events (PPAEs), including depression, suicide and suicidal ideation, learning and memory impairment, dementia, and schizophrenia (Andronis et al. 2020). Notably, these adverse events are often only detected late in drug development (e.g., Phase III clinical trials) or during pharmacovigilance (Phase IV), raising substantial safety concerns for patients and challenges for clinicians and pharmaceutical companies (Crowther 2013, Marques et al. 2024, Cook et al. 2014).

Rimonabant (SR141716A) was approved by the European Medicines Agency (EMA) in 2006 (Acomplia^®^), for the treatment of obesity, type II diabetes, and dyslipidaemia (EMA 2007, Doggrell 2008). Rimonabant’s primary mechanism of action was postulated to rely on high-affinity antagonism at cannabinoid receptor type 1 (CB_1_) (Rinaldi-Carmona et al. 1994), a G-protein-coupled receptor (GPCR) central to synaptic plasticity, mood, memory, and reward (Moreira and Crippa 2009, Cristino et al. 2020). By blocking CB_1_ signalling, rimonabant disrupts endocannabinoid-mediated regulation of appetite and energy homeostasis, supporting its clinical use as an anti-obesity agent (Doggrell 2008, Henness et al. 2006, Christensen et al. 2007). This drug also modulates glucose and lipid metabolism in peripheral tissues, paricularly adipose tissue, and acts on mitochondrial CB_1_ receptors, affecting cellular respiration and energy dynamics (Cristino et al. 2020). Notably, rimonabant has also been shown to interact with multiple receptor systems, including the µ-opioid (MOR) and the δ-opioid (DOR) receptors (Zádor et al. 2012, Zádor et al. 2014), peroxisome proliferator-activated receptors (PPARs), and transient receptor potential (TRP) channels, influencing neuroimmune signaling, sensory transduction, and inflammation (Moreira and Crippa 2009, Porcu et al. 2018). However, where available, the affinities for non-CB_1_ receptors are typically in the micromolar range (e.g., Zádor et al., 2015), more than 100 times lower than for the primary binding site (Rinaldi-Carmona et al., 1994), and the biological importance of such binding is equivocal.

Due to accumulating reports of suicides and suicidal ideation, rimonabant was withdrawn from the market in 2008 (Soler-Cedeno and Xi 2022). Despite this clinical evidence, the mechanisms underlying rimonabant’s neurotoxicity and its link to PPAEs remain poorly defined. Interestingly, emerging evidence points to epigenetic mechanisms (e.g., DNA methylation, histone modifications, non-coding RNAs) as pivotal in the onset and progression of psychiatric disorders (Fernández-Castillo and Martín-García 2022). For example, patients with major depressive disorder (MDD) have been shown to exhibit persistent reductions in trimethylated H3K4 (H3K4me3) levels on immune gene promoters, even after antidepressant treatment (Lin et al., 2022) (Tseng et al. 2023). Tsankova et al. (2007) showed that chronic social defeat stress-induced histone acetylation changes in the *nucleus accumbens* of mice correlated with depression-like behaviours (Tsankova et al. 2007). Moreover, genes within the endocannabinoid system (e.g., *cnr1*, *cnr2*, which code for CB_1_ and CB_2_ receptors, respectively) also undergo epigenetic changes (Basavarajappa and Subbanna 2022, Mehler 2008, D’Addario et al. 2013) that can disrupt normal neuronal function and promote susceptibility to psychiatric disorders and PPAEs (Gomes et al. 2020).

Building on the relevance of epigenetic modifications to neuronal function, we hypothesised that rimonabant could induce neurological adverse effects by modulating the epigenetic machinery. To address this hypothesis, we aimed to: (1) explore rimonabant’s ability to alter global DNA methylation and histone acetylation levels, as well as specific histone marks [including acetylated H3K9 (H3K9ac), and trimethylated H3K4 (H3K4me3) and H3K27 (H3K27me3)] in SH-SY5Y human neuroblastoma cells at pharmacologically relevant concentrations; (2) elucidate the pharmacological mechanisms underlying rimonabant-induced epigenetic changes; and (3) test rimonabant’s impact on epigenetic patterns ex vivo, in rat brain tissue. Notably, linking epigenetic-drug interactions to PPAEs may provide critical insights into the mechanisms of adverse events, enhancing patient safety and informing future drug safety assessments.

## Materials and methods

### Chemicals

Heat-inactivated fetal bovine serum (FBS), antibiotic (10 000 U/mL penicillin, 10 000 µg/mL streptomycin), Dulbecco’s Modified Eagle Medium (DMEM), phosphate-buffered saline (PBS) and Hanks’ balanced salt solution (HBSS) were obtained from Gibco Laboratories (UK). Normal horse serum (#S-2000) and TrueVIEW^®^ Autofluorescence Quenching kit with DAPI (#SP-8500) were acquired from Vector Laboratory Inc. (Newark, USA). The remaining reagents were purchased from Merck (Darmstadt, Germany).

Rimonabant, ≥ 98% purity (HPCL), was kindly provided by Sanofi S.A. (Montpellier, France). A 1 mM stock solution of the drug was prepared in dimethyl sulfoxide (DMSO). This solution was aliquoted and stored at - 20 °C until further use. Before cell exposure, this solution was sequentially diluted in HBSS to achieve a final DMSO concentration of less than 0.1 %, a concentration that has been previously demonstrated to be non-toxic to SH-SY5Y cells (Araújo et al. 2024).

### Cell culture

SH-SY5Y human neuroblastoma cells (ATCC^®^ CRL-2266^™^) were cultured in 25 cm^2^ cell culture flasks in DMEM supplemented with heat-inactivated 10% FBS and 1% of a 100 U/mL penicillin and 100 mg/mL streptomycin solution, at 37 °C, in a humidified atmosphere containing 5 % CO_2_. This cell line is one of the most widely used in neuroscience and neurotoxicology research due to its human origin, neuronal characteristics, and capacity to differentiate into mature neuron-like cells (Lopez-Suarez et al. 2022). SH-SY5Y cells also represent a suitable in vitro model for investigating epigenetic mechanisms, as they respond to various stimuli by altering histone acetylation and DNA methylation profiles (Trivedi et al. 2015), thus offering valuable insights into the epigenetic regulation of neuronal pathways. Cells were seeded at a density of 25,000 cells/cm^2^ in 6-well plates for genomic DNA, histone, and protein extraction, or 100 mm diameter Petri dishes for nuclei extraction.

After overnight adhesion, cells were exposed to either rimonabant for 24 and 96 h at two pharmacologically relevant concentrations (0.01 and 1 µM), a vehicle control (0.1 % DMSO), and a negative control (untreated cells). As a positive control, cells were exposed for 24 h to 100 nM trichostatin A (TSA), a well-characterised histone deacetylase (HDAC) inhibitor widely used to induce global histone acetylation in vitro, including in SH-SY5Y cells (Citraro et al. 2020, Rigby et al. 2012). For mechanistic purposes, a condition in which cells treated with 1 µM rimonabant for 96 h were further treated with 1 µM of the synthetic CB_1_ receptor agonist, ADB-FUBINACA, for additional 24h, was also considered.

### Sample preparation

At the designated timepoints, cells were collected using a cell scraper and centrifuged for 5 min at 1000 g using a ThermoScientific Multifuge X1R (Germany), after which the supernatant was discarded. The resulting pellet was used for DNA, histone, nuclei, or protein extraction, as described in the following sections:

#### Genomic DNA extraction

Genomic DNA was extracted from the pellet using the GRS Genomic DNA Kit - BroadRange (Grisp, Portugal), following the manufacturer’s instructions, with minor adaptations. Cell pellets were resuspended in 200 µL of phosphate-buffered saline (PBS), followed by the addition of 20 µL of proteinase K (10 mg/mL) and incubation at 60 °C for 5 min. Subsequently, 200 µL of Buffer BR2, meant for nuclear membrane disruption, was added, and the samples were incubated again at 60 °C for 5 min. To facilitate DNA binding, 200 µL of absolute ethanol was added to each sample, thoroughly mixed, and transferred to spin columns. Columns were centrifuged at 16,000 g for 1 min, and the retained DNA was subjected to sequential washes with 400 µL of Wash Buffer 1 and 600 µL of Wash Buffer 2, with centrifugation at 16,000 g for 1 min between each step. A final centrifugation at 16,000 g for 3 min was performed to remove residual wash buffer. DNA was eluted by incubating the columns with 100 µL of preheated Elution Buffer for 3 min at room temperature, followed by centrifugation at 16,000 g for 1 min. Purified genomic DNA samples were stored at - 80 °C until further use.

### Global DNA methylation

Global DNA methylation levels were quantified using the colorimetric Global DNA Methylation Assay Kit (5-Methyl Cytosine, ab233486, Abcam), according to the manufacturer’s protocol. This assay is based on an immunoassay principle wherein genomic DNA is immobilised onto specially treated strip wells with high DNA-binding affinity. The methylated cytosine residues (5-mC) are detected via a capture antibody specific for 5-mC, followed by a signal enhancer and colour developer solution. The resulting signal, measured colorimetrically, is directly proportional to the amount of 5-mC present in the DNA.

For each sample, 100 ng of purified genomic DNA (extracted as described in Sect.  “Genomic DNA extraction”) were mixed with 100 µL of Binding Solution in a 96-well plate and incubated at 37 °C for 1 h. After DNA binding, the wells were washed with 1X Wash Buffer and incubated with the 5-mC Detection Complex, followed by Developer Solution and Stop Solution to complete the colorimetric reaction. The absorbance was read at 450 nm in a BioTek Synergy™ HT microplate reader (BioTek Instruments, Inc., Winooski, Vermont, USA), and the % 5-mC was interpolated from a standard calibration curve (using standards ranging from 0 to 5 % of 5-mC content) to determine the percentage of methylated cytosines per total DNA.

#### Histone extraction

The pellet was resuspended in 500 µL of Triton Extraction Buffer (TEB, comprising PBS supplemented with 0.5% Triton X-100, 2 mM phenylmethylsulfonyl fluoride (PMSF), and 0.02 % NaN_3_, and the samples were kept on ice for 10 min. The samples were then centrifuged for 1 min at 10000 rpm in a MicroStar 12 centrifuge (VWR, Radnor, PA, USA) at 4 °C, and the supernatant was discarded. The pellet was then resuspended in 200 µL Histone Extraction Buffer (0.5 N HCl and 10 % glycerol) and kept on ice for additional 30 min. The suspension was then centrifuged at 12000 rpm (MicroStar 12 centrifuge, VWR, Radnor, PA, USA) for 5 min at 4 °C. After recovering the supernatant into a clean 1.5 mL tube, 600 µL of acetone were added to each sample, and the tubes were stored overnight at - 20 °C. On the following day, the samples were centrifuged again at 12000 rpm (MicroStar 12 centrifuge, VWR, Radnor, PA, USA) for 5 min, and the supernatant was evaporated under a gentle nitrogen flow. After the evaporation was completed, the pellet was dissolved in 50 µL of distilled water, and the samples were kept at - 80 °C for future use.

#### Nuclei extraction

The pellet was resuspended in 1 mL of lysis buffer (10 mM Tris-HCl, 10 mM NaCl, 15 mM MgCl_2_, 250 mM sucrose, 0.1 mM EGTA, and 0.5 % Triton-X 100) and vortexed for 30 s to promote cell lysis, before being incubated for 30 min on ice. Four millilitres of a sucrose cushion (30% sucrose, 10 mM Tris-HCl, 10 mM NaCl, and 3 mM MgCl_2_, pH 7.5) were added to the cell lysates, and these were centrifuged at 13,000 g (Multifuge X1R, Thermo Scientific, Darmstadt, Germany) for 10 min at 4 °C. The supernatants were discarded, and the pellets, containing the isolated nuclei, were washed with a cold solution of 10 mM Tris-HCl and 10 mM NaCl. The samples were then resuspended in 100 µL Nuclei Extraction Buffer (50 nM HEPES KOH at pH 7.5, 420 mM NaCl, 0.5 mM EDTA.Na_2_, 0.1 mM EGTA and 10 % glycerol), sonicated three times for 10 s each, and left on ice for 30 min. Following this cooling period, the samples were centrifuged at 16,000 g (Multifuge X1R, Thermo Scientific, Darmstadt, Germany) for 15 min, and the supernatants were collected into clean 1.5 mL tubes. The crude nuclear extracts were stored at - 80 °C for subsequent use.

#### Protein extraction

The pellet was resuspended in 100 µL of collecting buffer (20 mM HEPES, 250 mM sucrose, 10 mM KCl, 2 mM MgCl_2_, 1 mM EDTA, pH 7.5). At the time of the extraction, this buffer was supplemented with 2 mM dithiothreitol (DTT), 100 µM PMSF, and 1 µL/mL Protease Inhibitor Cocktail. The pellets were then disrupted by sonication with three pulses of 30 s intercalated with 30 s on ice. The samples were then stored at - 80 °C until further use.

### Protein quantification

The total protein amount in histone, nuclear, and total extracts was determined using the Detergent Compatible (DC) Protein Assay (Bio-Rad, Hercules, CA, USA) kit, according to the manufacturer’s instructions. The absorbances were measured at 750 nm in a BioTek Synergy™ HT (BioTek Instruments, Inc.) microplate reader and protein concentrations interpolated from a calibration curve using bovine serum albumin (BSA) as a standard.

### Global histone H3 and H4 acetylation

Global histone H3 and H4 acetylation levels were quantified using the fluorometric Histone H3 Total Acetylation Detection Fast Kit (ab131561, Abcam) and Histone H4 Total Acetylation Detection Fast Kit (ab131562, Abcam), respectively, following the manufacturer’s protocols. Both kits share a similar workflow with only minor variations.

Briefly, 200 ng of histone extract were added per well, in duplicate, to a 96-well plate configured to include a standard control supplied with the kit. The plate was incubated with Antibody Buffer for 1 h at 37 °C (or up to 2 h for the H3 acetylation assay). Following incubation, the plate was washed three times with 1× Wash Buffer and subsequently incubated with the Detection Solution for 1 h at 37 °C on an orbital shaker (100 rpm). After an additional wash step using 6× Wash Buffer, Fluoro-Development Solution was added to each well and incubated in the dark for up to 20 min. All reagents and working solutions were prepared according to the manufacturer’s instructions.

Fluorescence was measured using a Biotek Synergy HT microplate reader (BioTek Instruments, Inc., Winooski, Vermont, USA) with excitation and emission wavelengths of 530 nm and 590 nm, respectively. Results are expressed as the percentage of histone H3 or H4 acetylation relative to the vehicle control.

### Histone acetyltransferase activity

Histone acetyltransferase (HAT) activity was assessed using the colorimetric Histone Acetyltransferase Activity Assay Kit (ab65352, Abcam), according to the manufacturer’s protocol. This assay quantifies HAT activity by measuring the production of NADH, which results from the acetylation of a peptide substrate and subsequent release of CoA, enabling spectrophotometric detection at 440 nm through the reaction with a soluble tetrazolium dye. Absorbance was measured using a microplate reader (BioTek Instruments, Inc., Winooski, Vermont, USA). Results were presented as the activity of histone acetyltransferase, in µM/min.

### Histone deacetylase activity

Histone deacetylase (HDAC) activity was assessed using the fluorometric Histone Deacetylase Activity Assay Kit (ab156064, Abcam), according to the manufacturer’s instructions. This assay is based on the enzymatic deacetylation of a fluorescently labelled acetylated peptide substrate by HDACs, followed by cleavage of the resulting deacetylated product by a lysyl endopeptidase to release a fluorescent signal, which is directly proportional to HDAC activity. Fluorescence intensity was measured using a microplate reader (BioTek Instruments, Inc., Winooski, Vermont, USA) with excitation at 350–380 nm and emission at 440–460 nm. Results were presented as the activity of histone deacetylase, in µM/min.

### Western-blot

The expression of specific histone marks associated with PPAEs, namely H3K4me3, H3K27me3, and H3K9ac, was analysed by Western blot on total protein extracts from SH-SY5Y cells in the presence or absence of rimonabant. For Western blot, a total of 40 µg of each protein sample were denatured at 90 °C for 5 min in sodium dodecyl sulphate (SDS) sample buffer (0.25 M Tris-HCl, 50 % glycerol, 10 % SDS, 0.2 M DTT and 0.001 % bromophenol blue). The denatured samples were then separated via electrophoresis in 15 % SDS-polyacrylamide gels and subsequently transferred to polyvinylidene fluoride (PVDF) membranes (GE Healthcare, Pittsburgh, PA, USA), using a Trans-blot^®^ Turbo™ Blotting system (Bio-Rad, CA, USA), for 30 min, with the first 15 min at 100 V and the remaining 15 min at 150 V. The membranes were blocked with 5 % skimmed milk prepared in 0.05 % Tween 20 in phosphate-buffered saline (TPBS, pH 7.4) for 2 h at room temperature, on an orbital shaker. The membranes were then washed three times with TPBS for 10 min each. After washing, membranes were incubated overnight at 4 °C with the primary antibodies, rabbit anti-H3K9ac (1:1000), rabbit anti-H3K27me3 (1:1000) and rabbit anti-H3K4me3 (1:1000) from ThermoFisher Scientific (Harz, Germany), and mouse anti-β-actin (1:5000, Sigma-Aldrich, St Louis, MO, USA), using β-actin as a loading control. Primary antibodies were diluted in 1 % BSA prepared in TPBS and supplemented with 0.05 % sodium azide. The membranes were then washed again three times with TPBS for 10 min each and incubated with horseradish peroxidase (HRP)-conjugated secondary antibodies, anti-mouse immunoglobulin G (IgG, 1:20000) and anti-rabbit immunoglobulin (lgG 1:10000) from Advansta Inc, USA, both diluted in 1 % BSA prepared in TPBS, for 1 h at room temperature under mild stirring. The membranes were washed again in TPBS three times for 10 min, and the protein bands were detected using Clarity Western ECL Substrate (Advansta Inc., USA). Membranes were imaged using the molecular imager ChemiDoc™ XRS (Bio-Rad, Hercules, CA, USA). Band intensities in each lane were quantified using the Bio-Rad Image Lab (Version 6.1, Build 7, Bio-Rad, Hercules, CA, USA) and normalized against the intensities of the endogenous control β-actin, which was used as a housekeeping loading control to account for variations in total protein loading across samples. Notably, the use of β-actin as an endogenous control for the normalization of histone mark levels in total protein extracts has been previously described (Večeřa et al. 2018, Falcão-Holanda et al. 2023). Results were expressed as the fold-change relative to the vehicle control (cells treated with 0.1 % DMSO).

### Animals and experimental design

The Sprague-Dawley rat colony was purchased from Charles River, Italy, and the animals were subsequently bred and raised in the vivarium of the Faculty of Pharmacy, University of Belgrade, Serbia. The research was conducted in accordance with European Union guidelines (Directive 2010/63/EU) and approved by the Ethics Committee for Animal Experiments of the University of Belgrade – Faculty of Pharmacy, Serbia and the Ministry of Agriculture, Forestry and Water Management – Veterinary Directorate (323-07-09943/2018-05, 12/12/2018).

A total of 18 adult drug-naïve male Sprague-Dawley rats (*n* = 6 per group), weighing 250–300 g at the start of the experiment, were administered by oral gavage (10 ml/kg) either 3 mg/kg or 15 mg/kg rimonabant dispersed in a 0.25 % methylcellulose solution, or a 0.25 % methylcellulose solution alone, for 4 weeks. Whole brains were collected following anesthesia of the animals by intraperitoneal injection of a combination of ketamine (80 mg/kg) and xylazine (10 mg/kg), followed by perfusion with saline. Decapitation and brain removal from the skull were followed by three washes in PBS for 10 min each. The brains were then fixed in 4 % paraformaldehyde and placed in a series of sucrose solutions with increasing concentrations up to 30 %. Brain samples were then stored at - 80 °C in OLMOS cryoprotectant solution (0.95 % (*w*/*v*) Na_2_HPO_4_·2H_2_O, 0.212 % (*w*/*v*) NaH_2_PO_4_·H_2_O, 30 % (*w*/*v*) sucrose, 1 % (*w*/*v*) polyvinylpyrrolidone (PVP), 30 % (*v*/*v*) ethylene glycol) (Soares-Cardoso et al. 2024). For immunofluorescence, 35 μm-thick sections were serially sliced in the coronal plane from the brain samples using a vibratome, collected in 12-well plates and stored in OLMOS at - 20 °C until further processing.

### Analysis of epigenetic changes by immunofluorescence

Immunofluorescence was performed as described in previous publications (Neves et al. 2025, Sá et al. 2015). Briefly, for immunofluorescence-based labelling of the brain samples, a set of sections   (1/12) was thoroughly washed with PBS (four 15-min washes) to remove OLMOS. Subsequently, the sections underwent a 1 h blocking in a 5% normal horse serum solution, prepared in PBS with 0.1 % Triton X-100. The sections were then incubated with the following primary antibodies: mouse anti-5-methylcytosine nucleotide (33D3), MA5-38432; rabbit anti-H3K27me3 (G.299.10), MA5-11198 (1:500); rabbit anti-H3K4me3 (G.532.8), MA5-11199 (1:500) and rabbit anti-H3K9ac (J.924.2), MA5-11195 (1:500), all from Invitrogen (Carlsbad, CA, USA), for 72 h at 4 °C, in a free-floating manner. Following the incubation period, the sections underwent three 10 min washes, were mounted on a glass slide and allowed to dry-adhere to the glass surface. Upon re-hydration, the sections were exposed to the secondary antibodies, biotinylated anti-mouse IgG (H + L) BA-2001 (1:1000) with Streptavidin, Alexa Fluor™ 488, (S11223, Invitrogen, Carlsbad, CA, USA), and horse anti-mouse Dylight™ 594, (DI-2594, Vector Laboratories, CA, USA) (1:1000), protected from light, in a humidified chamber, for 1 h at room temperature. After three 10-min PBS washes to remove residual secondary antibodies, the sections were incubated with the TrueVIEW^®^ Autofluorescence Quenching kit with DAPI, following the supplier’s instructions. The slides were then mounted using Fluoroshield mounting media and stored at 4 °C.

**Fig. 1:**
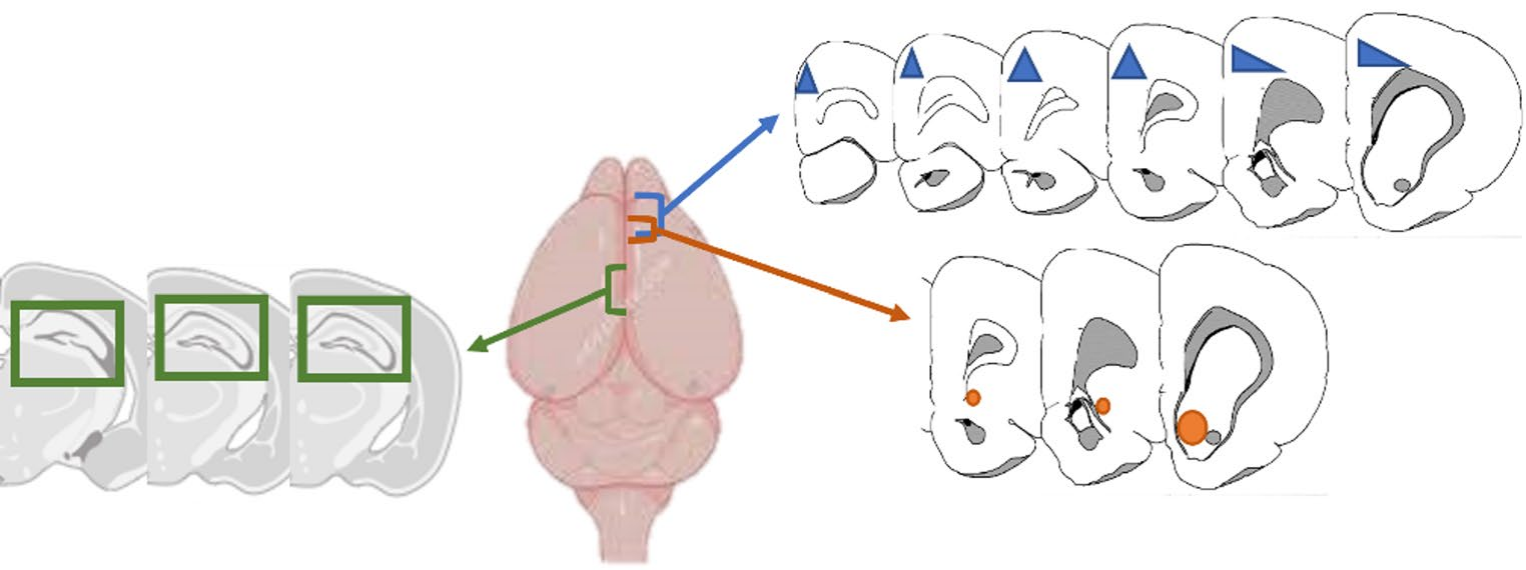
Representation of the brain areas of interest. The blue arrow and blue triangle illustrate the location and disposition of the prefrontal cortex (PFC). The orange arrow and circle represent the location and disposition of the *nucleus accumbens* (NAcc). Green arrow and squares indicate hippocampal formation location, composed of dentate gyrus (DG), Hilus (H) and CA1 and CA3. Representations not to scale

Images were acquired using a Carl Zeiss Axio Imager 2.0 microscope equipped with a colour camera and a computer supplied with the software Carl Zeiss AxioVision Rel. 4.8 (NY, USA). For each target protein, images were acquired with identical exposure times, gain, offsets, and slide z-stack distance. The filters’ excitation/emission wavelengths used were as follows: 365/445 nm, for DAPI; 470/525 nm, for Dylight and Alexa 488; and 565/620 nm, for Texas Red and Alexa 594. The following brain areas were analysed: prefrontal cortex (PFC), nucleus Accumbens (NAcc), and the hippocampal formation, which was further subdivided into dentate gyrus (DG), hilus (H), CA1, and CA3 (Fig. 1). The scanning and acquisition of images from these regions were executed manually, employing a 20x objective. Data are presented as the light intensity of each target per unit area (µm^2^).

### Statistical analysis

Cell culture assays were performed in duplicate. A minimum of 5 images were acquired for PFC and 4 of each distinct area of the hippocampal formation, while 3 images were captured for NAcc, per animal. For each animal, the mean of target intensity per total area and the sum of the number of cells per total area were assessed. Statistical analysis was performed using GraphPad Prism version 8.0.1 (GraphPad Software, CA, USA). The normality of each distribution was assessed using Anderson-Darling, D’Agostino-Pearson and Shapiro-Wilk normality tests, with consideration given to the acceptability of skewness and kurtosis values. Subsequently, group-level comparisons were performed using one-way ANOVA followed by Tukey’s or Dunnett’s post-hoc test. Statistical significance was considered at *p* < 0.05. Statistical levels were represented in the figures as follows: *p < 0.05, **p < 0.01, ****p < 0.001, and ****p < 0.0001.

## Results

### Rimonabant increased histone H3 and H4 acetylation levels in SH-SY5Y cells

**Fig. 2:**
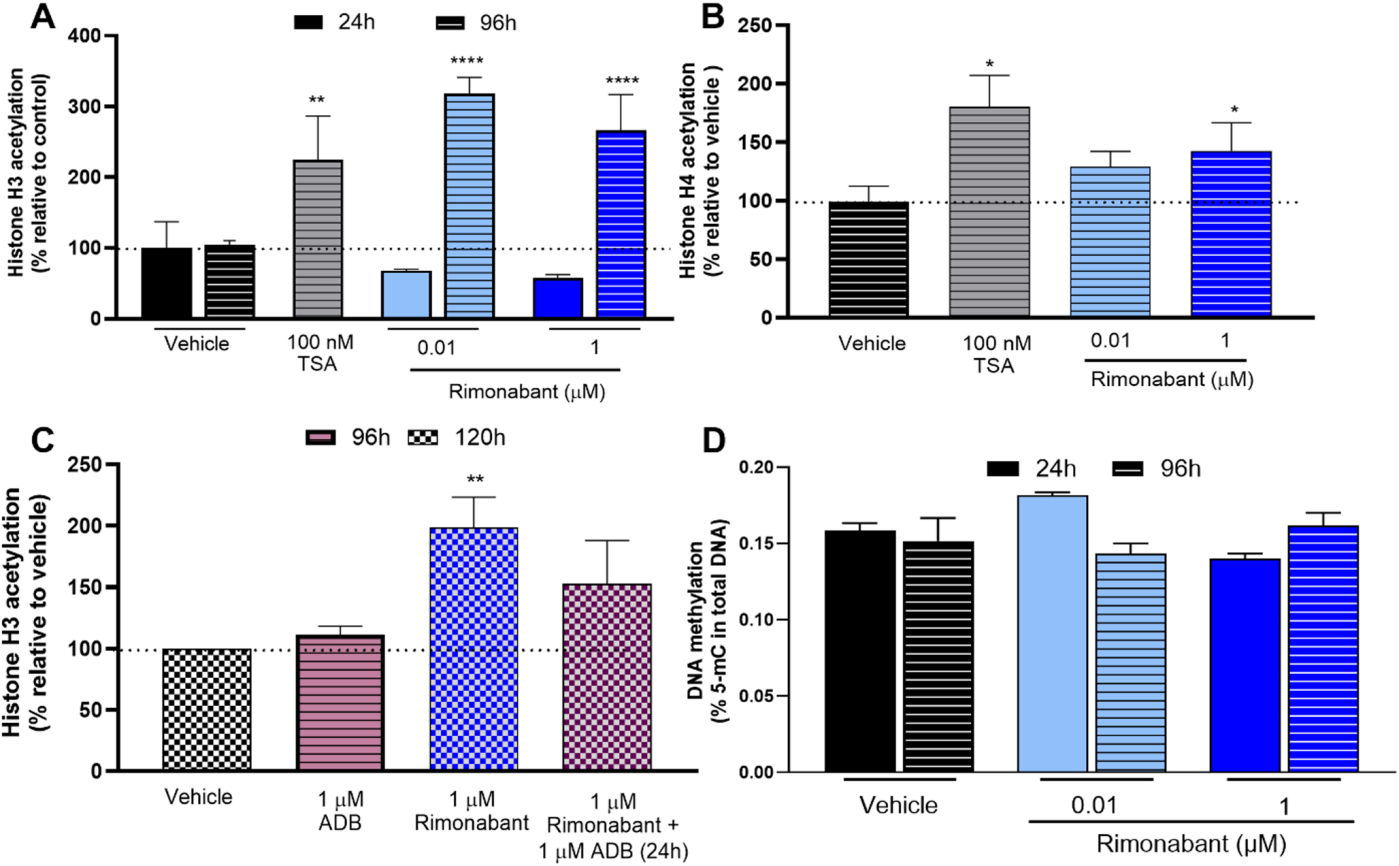
Assessment of rimonabant’s effects on global histone acetylation. SH-SY5Y cells were incubated for 96 h with 0.01 and 1µM rimonabant, 100 nM TSA or a vehicle control (0.1 % DMSO). (**A**) Global histone H3 and (**B**) H4 acetylation levels were quantified in histone extracts using commercially available kits (Abcam). (**C**) The potential involvement of the CB_1_ receptor in rimonabant-induced histone acetylation was assessed by incubating cells with 1 µM ADB-FUBINACA for additional 24 h, following cells’ incubation with rimonabant. (**D**) Global DNA methylation was assessed in DNA extracts using commercially available kits (Abcam). Each bar represents the mean ± S.E.M. for at least four independent experiments, performed in duplicate. * *p* < 0.05, ** *p* < 0.01, **** *p* < 0.0001 compared to vehicle control, using One-way ANOVA, followed by Dunnett´s post-test

Figure 2A and B show the effects of SH-SY5Y cell exposure to 0.01 and 1 µM rimonabant on histone H3 and H4 acetylation, respectively. Since no differences in histone H3 and H4 acetylation levels were observed between untreated cells and those exposed to the vehicle control (0.1% DMSO), it was confirmed that the vehicle had no impact on histone acetylation. Consequently, all data are expressed as a percentage relative to the vehicle control. In contrast, the positive control (100 nM TSA) induced a 2.2-fold and 1.8-fold increase in histone H3 and H4 acetylation, respectively, compared to the vehicle, thereby validating the sensitivity of the assay. As noted in Fig. 2A, 0.01 and 1 µM rimonabant increased global histone H3 acetylation by 3.2- and 2.7-fold, respectively, compared to the vehicle, after 96 h. Only 1 µM rimonabant increased histone H4 acetylation levels by 1.4-fold compared to the vehicle, after 96 h, as shown in Fig. 2B. The lowest concentration of rimonabant tested (0.01 µM) did not significantly alter this parameter.

To ascertain the potential involvement of the CB_1_ receptor in rimonabant-mediated increase in histone acetylation, we further incubated 96 h rimonabant-exposed cells with 1 µM ADB-FUBINACA (a synthetic CB_1_ receptor agonist) for an additional 24 h, for a total incubation of 120 h. As shown in Fig. 2C, ADB-FUBINACA markedly attenuated the rimonabant-induced significant increase in global histone H3 acetylation, reducing acetylation levels by approximately 46.6 % relative to rimonabant treatment alone. At the same time, 96 h incubation with ADB-FUBINACA alone presented no noteworthy change. This reversal supports the involvement of CB_1_ receptor blockade in the observed epigenetic changes, suggesting that receptor reactivation can partially counteract rimonabant’s effects on histone acetylation. This suggests both the involvement of the CB_1_ receptor in mediating rimonabant’s effects on global histone acetylation and the possibility of a cannabinoid receptor agonist reversing such modifications.

Notably, none of the rimonabant concentrations tested altered global DNA methylation levels (% 5-mC) in SH-SY5Y cells at any of the tested time points (Fig. 2D).

### Rimonabant decreased HDAC, but did not alter HAT activity in vitro

**Fig. 3:**
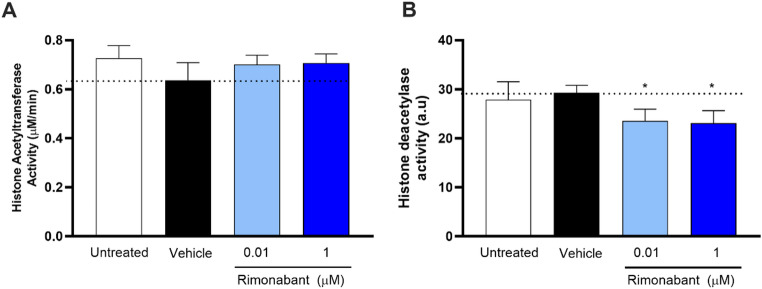
Evaluation of the rimonabant’s effects on histone acetyltransferase (HAT) and histone deacetylase (HDAC) activities. SH-SY5Y cells were incubated for 96 h with 0.01 and 1 µM rimonabant, and the activities of HAT (**A**) and HDAC (**B**) were measured in nuclear extracts using commercially available colorimetric kits (Abcam). Each bar represents the mean ± S.E.M. for at least four independent experiments, performed in duplicate. * *p* < 0.05, compared to vehicle control (0.1 % DMSO) using One-way ANOVA, followed by Dunnett´s post-test

To further explore the mechanisms underlying rimonabant-induced histone acetylation, we evaluated its impact on the enzymes responsible for histone acetylation (HATs) and deacetylation (HDACs). Considering that rimonabant’s effects on this parameter were only observed after 96 h, we selected this time point for the subsequent assays. As observed in Fig. 3A, rimonabant did not alter HAT activity at either of the concentrations tested (0.01 µM and 1 µM), compared to the vehicle control. However, as presented in Fig. 3B, rimonabant decreased HDAC activity by 19.6 % and 23.1 % at 0.01 µM and 1 µM, respectively, compared to the vehicle, suggesting reduced HDAC activity as a potential mechanism underlying rimonabant’s impact on histone acetylation.

### Rimonabant affects the expression of specific histone marks in vitro

**Fig. 4:**
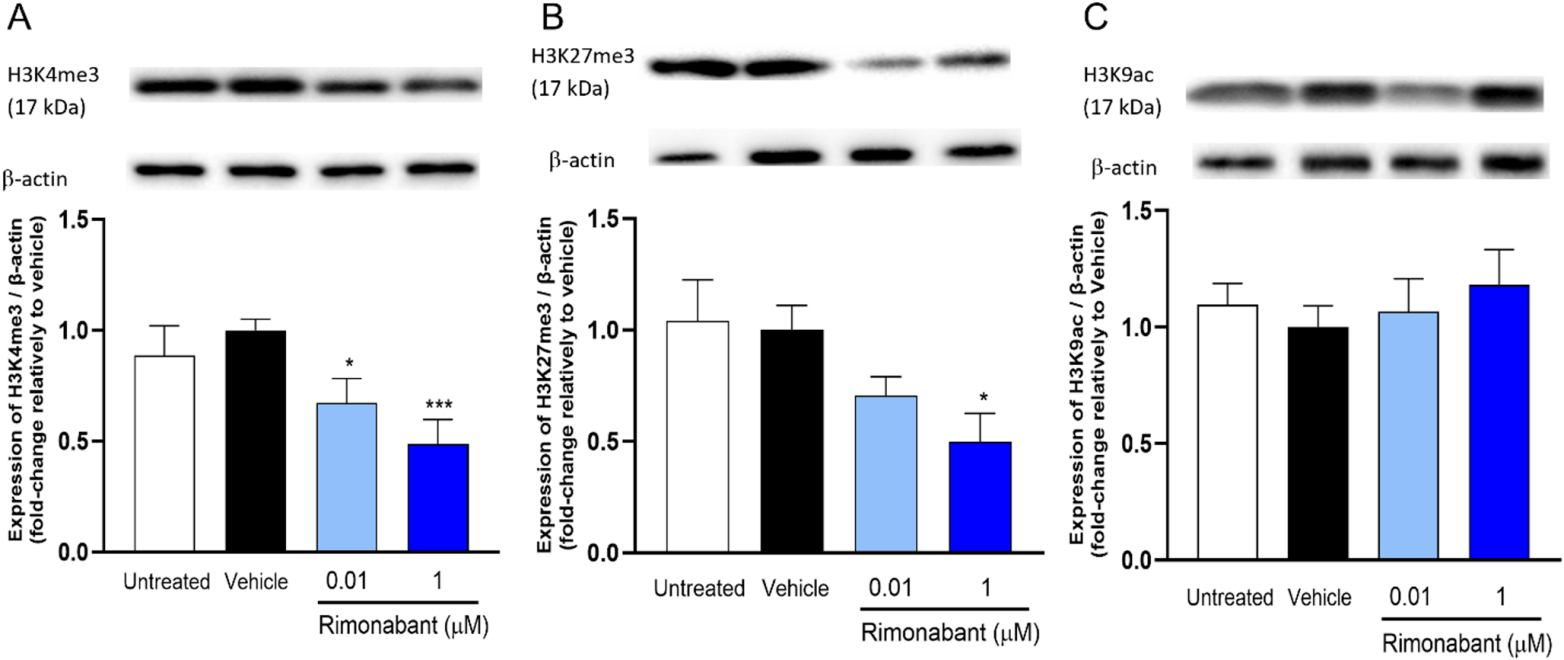
Expression of specific histone modifications associated with PPAEs. The SH-SY5Y cells were incubated for 96 h with 0.01 and 1 µM rimonabant, and the expression of H3K4me3 (**A**), H3K27me3 (**B**), and H3K9ac (**C**) was assessed by Western Blot. Their expression was normalized by the amount of β-actin in each condition and graphically represented as the percentage of expression relative to the vehicle (0.1 % DMSO). Representative protein bands are also shown. Each bar represents the mean ± S.E.M. for at least four independent experiments. * *p* < 0.05, *** *p* < 0.001, compared to vehicle (0.1 % DMSO), using a one-way ANOVA, followed by Dunnett’s post-test

We also assessed the impact of rimonabant on the expression of specific histone marks previously associated with PPAEs, in SH-SY5Y cells. As shown in Fig. 4, rimonabant decreased the expression of H3K4me3 (Fig. 4A) and H3K27me3 (Fig. 4B) in SH-SY5Y compared to vehicle-treated cells. Specifically, both concentrations of rimonabant tested (0.01 and 1 µM) decreased the expression of H3K4me3 by 32.7 % and 51.2 %, respectively, compared to the vehicle (Fig. 4A). Moreover, 1 µM rimonabant reduced H3K27me3 expression by approximately 50.2 % relative to the vehicle (Fig. 4B). No statistically significant changes were detected in H3K9ac expression (Fig. 4C) relative to vehicle-treated cells.

### Impact of rimonabant on epigenetic marks in vivo

**Fig. 5:**
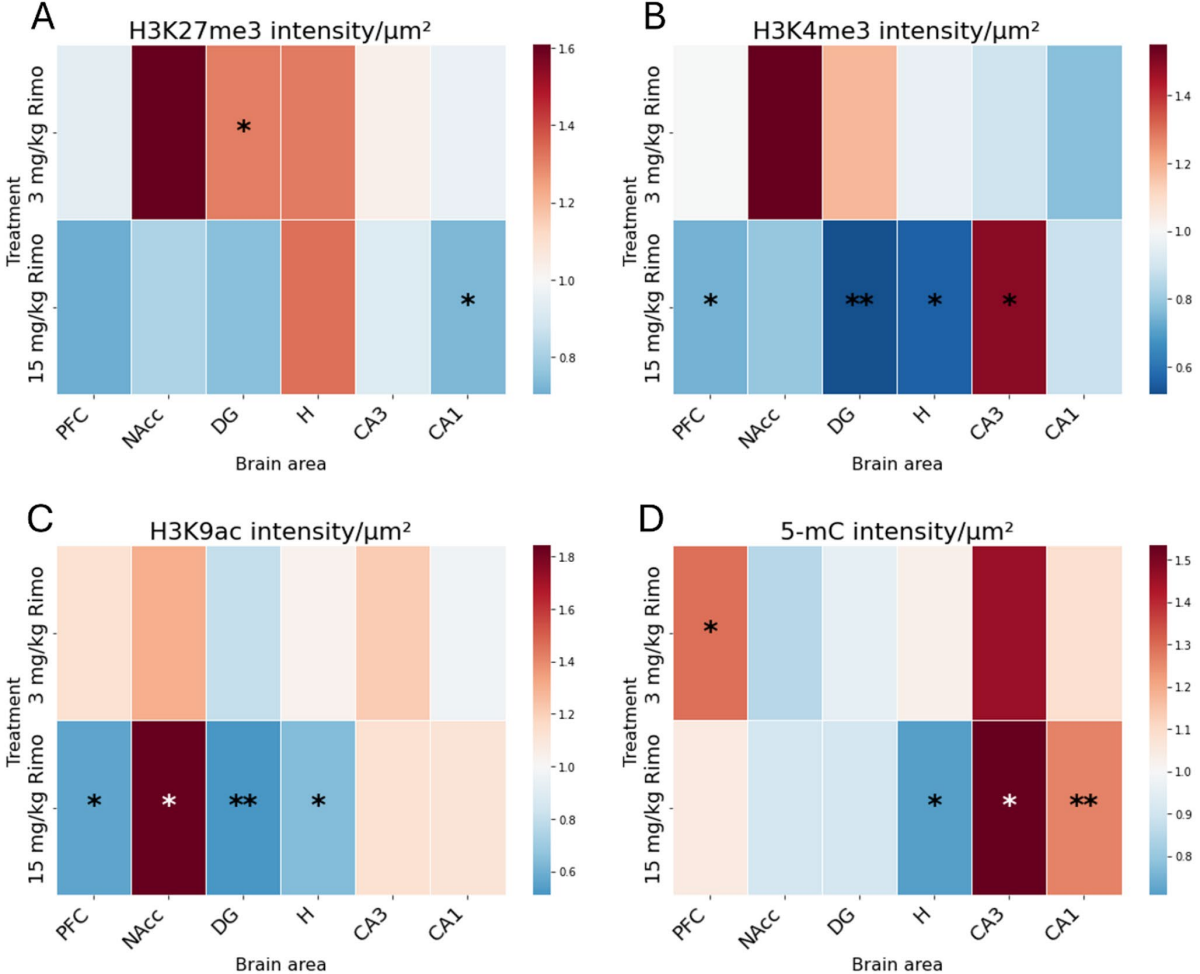
Heatmap representing the variation H3K27me3 (**A**), H3K4me3 (**B**), H3K9ac (**C**) and 5-mC (**D**) intensities (intensity/µm^2^) across different brain regions following rimonabant administration. Sprague-Dawley rats were administered 3 or 15 mg/kg rimonabant through oral gavage for 4 weeks. Each IF contained at least 5 animals per treatment group, and each region was photographed 3-7 times per animal. In the heatmaps presented, each rectangle represents the mean fluorescence intensity per square micrometre, normalised to the control, colour-coded according to the scale bar on the right. Warmer colours (red) indicate higher intensities, while cooler colours (blue) indicate lower intensities. * *p* < 0.05, ** *p* < 0.01, *** *p* < 0.001, **** *p* < 0.0001. PFC: prefrontal cortex; NAcc: nucleus Accumbens; DG: dentate gyrus; H: Hilus; CA3; CA1

The ability of rimonabant to change epigenetic marks was further explored in a rodent model following administration of 3 or 15 mg/kg rimonabant for 4 weeks. To facilitate the visualization of multiple epigenetic endpoints across different brain regions, the data presented in Fig. 5 are displayed as heatmaps, allowing a compact and comparative overview of regional and dose-specific changes. The corresponding quantitative data for each marker and brain region are available in the supplementary information. Overall, the most prominent rimonabant-induced epigenetic alterations were observed in the dentate gyrus (DG), prefrontal cortex (PFC), and hilus, with histone acetylation (H3K9ac) and H3K4me3 methylation showing the most consistent and widespread modulation across treatment conditions. The variation of the H3K27me3, shown in supplementary image 1, is represented in Fig. 5. The treatment with 3 mg/kg rimonabant increased the H3K27me3 by 60 % in the DG. In turn, the higher dose tested (15 mg/kg) only significantly decreased the levels of this mark in the CA1 by 27 % (Fig. 5A). This higher rimonabant dose further decreased H3K4me3 levels by 26 % in the PFC, 48 % in the DG and 44 % in the Hilus, while increasing this mark by 49 % in the CA3 (Fig. 5B). Similarly, 15 mg/kg rimonabant decreased H3K9ac levels by 44 % in the PFC, 49 % in the DG, and 36 % in the hilus. Notably, the same dose increased H3K9ac intensity/area by 84 % in the NAcc, compared to the control (Fig. 5C).

As noted in Fig. 5D, rimonabant further affected 5-mC levels in rat brains. Specifically, administration of 3 mg/kg rimonabant increased 5-mC intensity/area by 29 % in the PFC. In turn, 15 mg/kg rimonabant decreased 5-mC levels in the hilus by 29 %, while increasing them in the CA1 and CA3 by 26 % and 53 % respectively.

## Discussion

In line with the first objective of this study, we demonstrated that, while global DNA methylation levels (% 5-mC) remained unchanged following exposure to pharmacologically relevant concentrations of rimonabant (0.01 and 1 µM), the drug significantly increased global histone H3 and H4 acetylation. This range of concentrations has been previously reported to exert biologically meaningful but not cytotoxic effects, including modulation of signaling cascades and epigenetic regulators, in neuronal (including SH-SY5Y) and peripheral models (Foster et al. 2012, Zádor et al. 2015). For instance, 100 nM rimonabant was shown to alter mitogen-activated protein kinase (MAPK) activity and induce the expression of adipogenic markers, such as aP2 (adipocyte Protein 2), in 3T3-F442A cells (Gary-Bobo et al. 2006), while concentrations ≤ 1 µM selectively modulated CB_1_ receptor activity without major cytotoxicity (Zádor et al. 2014).

Beyond global acetylation changes, rimonabant modulated specific histone marks previously associated with PPAEs, including H3K4me3, and H3K27me3 (Bagot et al. 2014, Park et al. 2022). Histone acetylation plays a central role in the regulation of gene expression by relaxing chromatin structure and enhancing DNA accessibility to transcriptional machinery (Lopez-Suarez et al. 2022, Kim et al. 2009). As such, the observed rimonabant-induced increase in histone acetylation may facilitate the inappropriate activation of genes involved in key processes such as synaptic remodeling, stress reactivity, and neurodevelopment. Dysregulation of these pathways has been repeatedly implicated in the pathogenesis of neurological and psychiatric disorders (Citraro et al. 2020, McEwen et al. 2012).

Among the histone methylation marks evaluated, H3K4me3 is widely recognized as a transcription-promoting modification enriched at the promoters of genes involved in neuronal plasticity, immune signaling, and synaptic function (Dong et al. 2015, Gates et al. 2017). Decreased H3K4me3 has been observed in the cerebral cortex of patients with major depressive disorder (MDD), schizophrenia, and autism (Shen et al. 2014), as well as at synapsin gene promoters in postmortem samples from individuals with MDD and bipolar disorder (Melas et al. 2021). Notably, Tseng et al. (2023) reported reduced H3K4me3 levels at immune gene promoters (e.g., TNFAIP3, TLR4, TNIP2) in peripheral blood mononuclear cells from MDD patients, with these changes persisting despite antidepressant treatment (Tseng et al. 2023). Such persistent hypomethylation may reflect stable epigenetic traits that contribute to long-term vulnerability to psychiatric disorders (Csoka and Szyf 2009).

H3K27me3 is a repressive histone mark essential for the silencing of developmental and stress-responsive genes (Falcão-Holanda et al. 2023, Bogliotti and Ross 2012). In our model, exposure to 1 µM rimonabant significantly reduced H3K27me3 levels, suggesting disruption of transcriptional repression mechanisms. Similar reductions have been associated with emotional dysregulation, increased stress susceptibility, and depression-like phenotypes in preclinical studies (Melas et al. 2021, Hunter et al. 2009). For example, reduced H3K27me3 has been observed in stress-exposed hippocampal regions and linked to enhanced fear memory retrieval and maladaptive behavioral responses (Ell et al. 2024). These findings suggest that rimonabant-induced downregulation of H3K27me3 may compromise stress-adaptive transcriptional programs and contribute to affective instability.

Although H3K9ac levels were not significantly altered in vitro, this mark remains a relevant indicator of transcriptionally active chromatin and has been implicated in PPAEs. Region-specific changes in H3K9ac were previously observed in response to cannabinoid exposure in vivo. Pastrana-Trejo et al. (2021) reported increased H3K9ac in the cerebral cortex but decreased levels in the hypothalamus following administration of cannabidiol. Notably, decreased H3K9ac was associated with depressive-like behavior, while increased levels were linked to anxiety-like phenotypes (Melas et al. 2021). These data suggest that H3K9ac modulation is region- and context-dependent, which may explain the absence of detectable changes in the present in vitro system. Nonetheless, this reinforces the need for complementary in vivo investigations to capture region-specific epigenetic dynamics.

Taken together, these findings indicate that rimonabant exerts selective epigenetic alterations in vitro, particularly through increased histone acetylation and reduced levels of H3K4me3 and H3K27me3. These changes may underlie dysregulated gene expression profiles implicated in the pathogenesis of psychiatric and psychological adverse effects.

A mechanistically important finding was that co-incubation with ADB-FUBINACA, a potent synthetic CB_1_ receptor agonist, attenuated the rimonabant-induced increase in histone H3 acetylation. This supports two key conclusions: (1) rimonabant-induced histone acetylation is, at least partially, mediated through CB_1_ receptor antagonism, and (2) such epigenetic changes may be pharmacologically reversible. The ability to modulate these alterations is particularly relevant in the context of drug-induced adverse effects associated with aberrant histone acetylation.

CB_1_ is the most abundant G-protein-coupled receptor in the mammalian brain and is widely distributed across regions such as the hippocampus, amygdala, and prefrontal cortex. In these areas, CB_1_ modulates synaptic transmission via retrograde endocannabinoid signaling, playing a crucial role in emotion, cognition, and stress responses (Cristino et al. 2020, Szutorisz and Hurd 2016). Disruption of CB_1_ signaling, whether by pharmacological blockade or genetic deletion, has been associated with mood dysregulation, impaired memory, and altered stress reactivity (Moreira and Crippa 2009, Gomes et al. 2020, Mechoulam and Parker 2013), reinforcing its relevance in both therapeutic and adverse neuropsychological outcomes.

Endocannabinoid signaling has also been implicated in the regulation of epigenetic enzymes, including those responsible for histone modifications such as acetylation, methylation, and phosphorylation. In particular, CB_1_ receptor activation has been shown to influence gene transcription by modulating the activity of histone-modifying enzymes (D’Addario et al. 2013), providing a mechanistic link between CB_1_-mediated signaling and chromatin remodeling.

Our work shows that rimonabant-induced histone acetylation appears to result primarily from a reduction in HDAC activity, rather than increased HAT function. This is supported by the observed decrease in HDAC activity following rimonabant exposure, without changes in HAT activity. These findings suggest that CB_1_ antagonism by rimonabant may increase histone acetylation by inhibiting deacetylation processes. This interpretation contrasts with a previous study reporting that rimonabant reduced HAT activity in HCT116 and SW48 cells (Proto et al. 2017). However, it is important to note that in that study, rimonabant was tested at a supra-pharmacologically relevant concentration (10 µM), which also resulted in antiproliferative effects, potentially confounding the interpretation of epigenetic outcomes.

Notably, decreased HDAC activity and the resulting accumulation of acetylated histones have been implicated in neuropsychiatric and cognitive dysfunctions. For example, elevated HDAC activity in the hippocampus has been associated with reduced histone acetylation and cognitive deficits in schizophrenia models (Večeřa et al. 2018), while abnormal HDAC expression has been proposed as a contributing factor in the epigenetic dysregulation underlying psychiatric conditions (Verhoeff and Dhillon 2020). Together, our findings suggest that HDAC inhibition may represent a key mechanism in rimonabant-induced histone hyperacetylation, potentially linking CB_1_ receptor blockade to transcriptional dysregulation relevant to PPAEs.

Our ex vivo data revealed region- and dose-dependent alterations in histone acetylation and methylation across multiple brain regions, with the DG, PFC, and hilus being particularly sensitive to the effects of rimonabant. Notably, H3K4me3 levels were consistently reduced in both SH-SY5Y cells and in rat brain regions such as the PFC, DG, and hilus following high-dose rimonabant, suggesting a convergent mechanism of transcriptional repression. This finding is of particular interest given that reductions in H3K4me3 have been implicated in the pathophysiology of depression and schizophrenia, and are known to impair the expression of genes involved in neuronal plasticity and synaptic function (Bagot et al. 2014, Dong et al. 2015, Shen et al. 2014).

Similarly, H3K27me3 was downregulated in vitro and in hippocampal regions such as CA1, supporting the hypothesis that rimonabant disrupts transcriptional silencing mechanisms involved in emotional regulation. Notably, rimonabant-induced modulation of H3K27me3 appeared to be dose-dependent: 3 mg/kg increased H3K27me3 in the NAcc, whereas 15 mg/kg decreased this mark in CA1. These divergent effects are consistent with previous findings showing that acute stress and psychotropic drug exposure can reduce H3K27me3 levels in hippocampal and limbic areas (Tsankova et al. 2007). As H3K27me3 is a key repressive mark regulating developmental and stress-responsive genes (Tseng et al. 2023, Bogliotti and Ross 2012), its downregulation in regions such as the DG and CA1 has been associated with altered emotional processing and maladaptive fear memory retrieval (Melas et al. 2021, Hunter et al. 2009, Ell et al. 2024). These data suggest that region-specific loss of H3K27me3 may contribute to the behavioural disturbances associated with rimonabant.

Although no significant changes in H3K9ac (a histone mark typically associated with transcriptional activation) were observed in vitro, our ex vivo data showed marked reductions in the PFC, DG, hilus, and NAcc following rimonabant administration. This repressive pattern suggests that rimonabant may promote epigenetic silencing in cortical and limbic regions, potentially contributing to behavioural outcomes such as emotional blunting, cognitive impairment, and anxiety (Tsankova et al. 2007). While these findings appear to diverge from our in vitro results, such discrepancies likely reflect the regional and cellular complexity of the intact brain. The observed modulation of H3K9ac across functionally distinct areas highlights the importance of considering region-specific vulnerability when investigating the neuroepigenetic consequences of pharmacological exposure.

Global DNA methylation, as indicated by 5-mC levels, remained unchanged in SH-SY5Y cells; however, rimonabant significantly altered % 5-mC ex vivo in a dose- and region-specific manner. Specifically, rimonabant increased 5-mC in the CA1 and CA3 regions while decreasing it in the hilus. These findings suggest localized changes in DNA methylation across hippocampal subregions, which may reflect adaptive or maladaptive transcriptional responses depending on the region. Such region-specific patterns of DNA methylation are consistent with prior studies showing that early-life adversity can alter 5-mC levels in parvalbumin-positive interneurons of the PFC in a sex-dependent manner, with lasting effects on affective behaviour and neurodevelopment (Noel et al. 2024). Similarly, environmentally relevant exposures to common pharmaceuticals such as ciprofloxacin and paracetamol disrupted 5-mC levels in zebrafish embryos, along with neurodevelopmental and behavioural disturbances (Nogueira et al. 2019). These examples underscore the sensitivity of DNA methylation to both pharmacological and environmental challenges, supporting the interpretation that rimonabant-induced alterations in 5-mC may reflect transcriptional dysregulation relevant to its psychiatric and psychological adverse effects.

Given the essential role of epigenetic plasticity in shaping behavioural outcomes, our findings anticipate that these epigenetic modifications may contribute to the psychiatric side effects associated with rimonabant treatment, particularly under chronic exposure. Moreover, the bidirectional nature of rimonabant’s epigenetic actions, where the same mark may increase in one region and decrease in another, underscores the need to account for regional specificity in preclinical assessments of psychiatric drug safety. The observation that rimonabant suppresses high constitutive activity at both mitogen-activated protein kinase and adenylyl cyclase levels in Chinese hamster ovary cells transfected with human CB_1_ indicates that rimonabant may act not as a classical antagonist, but as an inverse agonist for autoactivated CB_1_ (Bouaboula et al. 1997). This may further explain its propensity to induce bidirectional actions. Altogether, these results strengthen the hypothesis that rimonabant-induced PPAEs arise not from global epigenetic dysregulation, but from selective disruption of neuroepigenetic homeostasis within key emotional and cognitive pathways, notably the PFC and hippocampus.

The divergence observed between in vitro and ex vivo outcomes, particularly in global DNA methylation, highlights the limitations of monoculture systems in capturing the full complexity of neuroepigenetic regulation. Although SH-SY5Y cells provide a convenient and well-characterized model for initial screening, they lack the cellular heterogeneity, regional architecture, and circuit-level integration of the intact brain, all of which are essential for modelling region-specific epigenetic responses. This is especially relevant for DNA methylation, a long-term and context-dependent epigenetic mechanism known to be influenced by intercellular signalling, stress exposure, and neuronal activity, factors largely absent or reduced in simplified culture systems.

Overall, this work contributes to a deeper understanding of the molecular mechanisms underlying cannabinoid-based therapies and their potential adverse effects. It underscores the importance of integrating epigenetic endpoints into neuropharmacological safety assessments, particularly for drugs targeting the endocannabinoid system. Future studies should investigate the reversibility of these changes and their behavioural correlates, potentially informing strategies for early risk assessment and safer drug development in neuropsychopharmacology.

## Supplementary Information

Below is the link to the electronic supplementary material.

Supplementary Material 1
