# Supplementary material for: Therapeutically relevant rimonabant exposure drives epigenetic remodeling in neuronal cells and rat brain tissue
Source: Arch Toxicol. 2026 Apr 17;100(7):3159–72. doi: 10.1007/s00204-026-04369-0 (PMC13309441; doi:10.1007/s00204-026-04369-0)
Supplement: Supplementary file 1 — Supplementary Material 1 [file 204_2026_4369_MOESM1_ESM.docx]

**Rimonabant-mediated epigenetic remodelling in a neuronal cell line and rat brain tissue at therapeutically relevant concentrations**

**- Supplementary Information -**

Sandra I Marques ^a, b^, Matilde Barreiras de Moura ^a, b^, Federica Panza ^a, b^, Catarina Pereira-Teixeira ^a, b^, Vladimir Stevanović ^c^, Aleksandra Kovačević ^c^, Miroslav Savić ^c^, Helena Carmo ^a, b^, Susana I Sá ^d,e^, Félix Carvalho ^a, b, #^, João Pedro Silva ^a, b, #^

^a^UCIBIO – Applied Molecular Biosciences Unit, Laboratory of Toxicology, Faculty of Pharmacy, University of Porto, 4050-313 Porto, Portugal;

^b^i4HB – Institute for Health and Bioeconomy, Faculty of Pharmacy, University of Porto, 4050-313 Porto, Portugal;

^c^Department of Pharmacology, Faculty of Pharmacy, University of Belgrade, Serbia;

^d^Unit of Anatomy, Department of Biomedicine, Faculty of Medicine, University of Porto; ^e^CINTESIS@RISE, Faculty of Medicine, University of Porto, Portugal

*Co-senior and corresponding authors:

João P. Silva, Félix Carvalho,

Associate Laboratory i4HB - Institute for Health and Bioeconomy, UCIBIO, Laboratory of Toxicology, Department of Biological Sciences, Faculty of Pharmacy, University of Porto, 4050-313, Porto, Portugal. E-mails: [jpmsilva@ff.up.pt](mailto:jpmsilva@ff.up.pt) (JPS), [felixdc@ff.up.pt](mailto:felixdc@ff.up.pt) (FC). Tel: +351 220428600


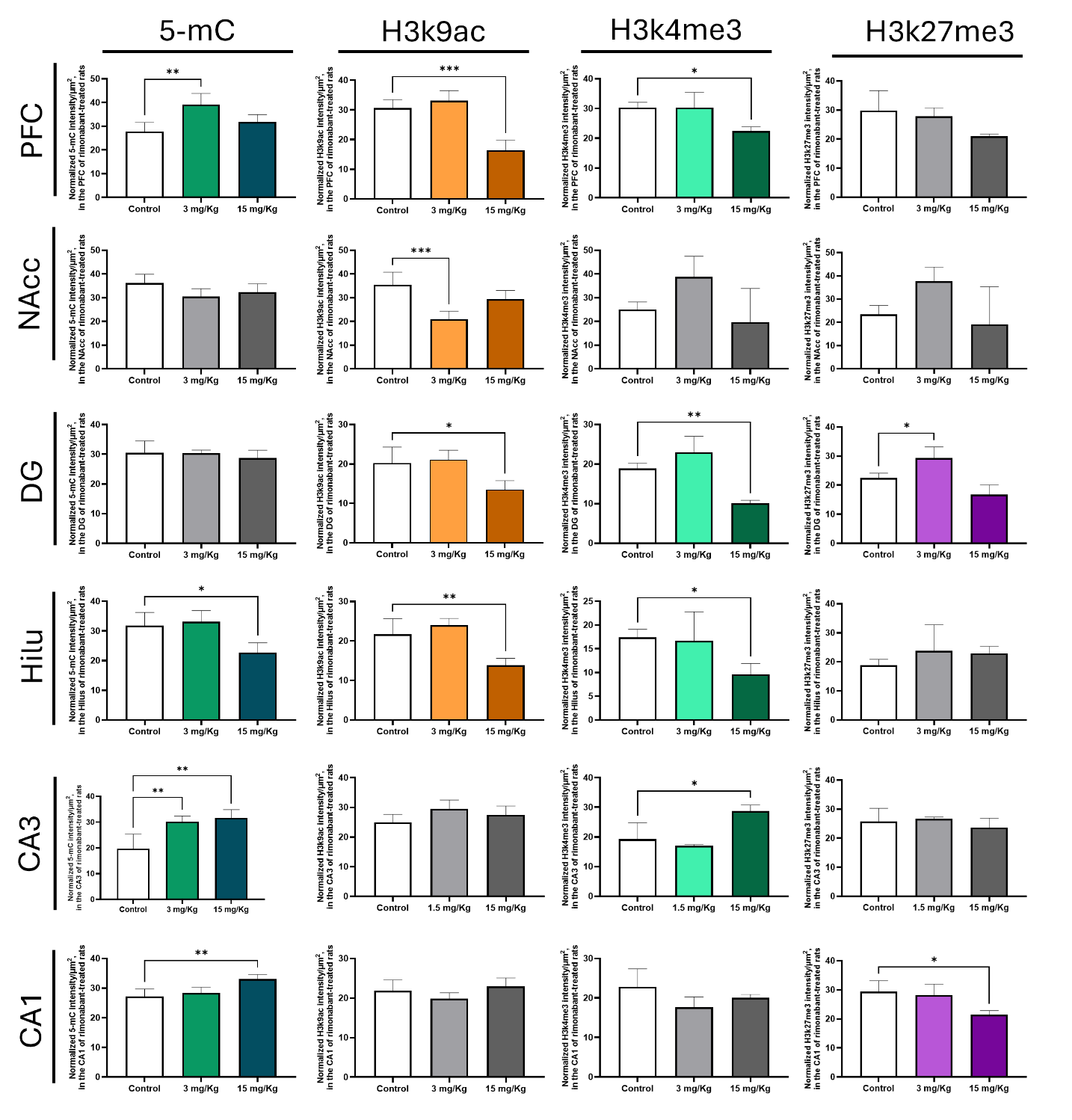


**Figure SI-1 - Effects of rimonabant [Data from Figure 5 presented the expression in intensity per µm^2^ of 5-mC, H3K9ac, H3K4me3 and H3K27me3].** Sprague-Dawley rats were administered 3 or 15 mg/kg rimonabant through oral gavage for 4 weeks. Levels of expression in PFC, NAcc, DG, Hilus, CA3 and CA1, were evaluated after 4 weeks of treatment. Each IF contained at least 5 animals per treatment group, and each region was photographed 3-7 times per animal. PFC: prefrontal cortex; NAcc: nucleus Accumbens; DG: dentate gyrus; H: Hilus; CA3; CA1. * < p 0.05, ** p < 0.01, *** < p 0.001, compared to the vehicle, using one-way ANOVA followed by Dunnett’s test.
